# The nursing practice environment and hospital sociotechnical complexity: a mixed-methods study

**DOI:** 10.1590/0034-7167-2023-0315

**Published:** 2024-12-16

**Authors:** Caren de Oliveira Riboldi, Renata Cristina Gasparino, Wiliam Wegner, Eder Henriqson, Tarcísio Abreu Saurin, Ana Maria Müller de Magalhães

**Affiliations:** I Hospital de Clínicas de Porto Alegre. Porto Alegre, Rio Grande do Sul, Brazil; II Universidade Estadual de Campinas. Campinas, São Paulo, Brazil; III Universidade Federal do Rio Grande do Sul. Porto Alegre, Rio Grande do Sul, Brazil; IV Pontifícia Universidade Católica do Rio Grande do Sul. Porto Alegre, Rio Grande do Sul, Brazil

**Keywords:** Complexity Analysis, Health Facility Environment, Nursing, Patient Safety, Quality of Health Care., Análisis de Complejidad, Ambiente de Instituciones de Salud, Enfermería, Seguridad del Paciente, Calidad de la Atención de Salud.

## Abstract

**Objectives:**

to analyze the relationship between the nursing practice environment and hospital sociotechnical complexity as perceived by nurses.

**Methods:**

a sequential explanatory mixed-methods study was conducted in a hospital in southern Brazil. The Brazilian version of the Practice Environment Scale-Nursing Work Index and the Complexity Characterization Questionnaire were administered to 132 nurses. Subsequently, semi-structured interviews were conducted with 18 participants, and the data were subjected to thematic analysis. Data integration was achieved through a connection approach.

**Results:**

the nursing practice environment was found to be favorable, except in the subscale concerning Staffing and Resource Adequacy, where complexity was present in the activities. The three emerging categories explained human and technical aspects related to complexity in the practice environment, quality of care, and patient safety. Unexpected variability was inversely correlated with the practice environment.

**Conclusions:**

the study results indicate a relationship between these constructs, with implications for the quality and the safety of care.

## RESUMEN

**Objetivos:**

analizar la relación entre el entorno de práctica profesional de enfermería y la complejidad sociotécnica hospitalaria desde la percepción de los enfermeros.

**Métodos:**

estudio explicativo secuencial de métodos mixtos realizado en un hospital del sur de Brasil. Se aplicaron la versión brasileña de la *Practice Environment Scale-Nursing Work Index* y el Cuestionario de Caracterización de la Complejidad a 132 enfermeros. Posteriormente, se realizaron entrevistas semiestructuradas con 18 participantes, sometidas a análisis temático. La integración de los datos se llevó a cabo mediante conexión.

**Resultados:**

el entorno de práctica se mostró favorable, excepto en la subescala de Adecuación de personal y recursos, donde se observó complejidad en las actividades. Las tres categorías emergentes explicaron aspectos humanos y técnicos relacionados con la complejidad en el entorno de práctica, la calidad del cuidado y la seguridad del paciente. La variabilidad inesperada se correlacionó inversamente con el entorno de práctica.

**Conclusiones:**

los resultados del estudio indican una relación entre estos constructos, con implicaciones para la calidad y la seguridad del cuidado.

## INTRODUCTION

Large hospital organizations with high levels of care requirements possess characteristics that tend to amplify their complexity, such as a diversity of pathologies and professional specialties, patients with multiple chronic conditions and clinical severity, demand for hard-to-replace services, and the use of innovative diagnostic and therapeutic technologies, among others. Moreover, all these elements do not behave in an isolated or linear manner, as they are subject to the stimuli, resources, information, and feedback present in the environment where they are embedded^(1)^.

In nursing, the place where care actions occur and these elements interact is called the professional practice environment, which is defined by the presence or absence of characteristics that facilitate or restrict the development of activities. Among the aspects that make an environment favorable for work are professional autonomy, decentralized decision-making, high-quality clinical care, participative leadership, opportunities for training and career advancement, and collaborative collegial relationships^(2)^.

Healthcare services are considered highly complex sociotechnical systems (STS), composed of interdependent social elements (people and their relationships) and technical elements (e.g., equipment and software)^(3,4)^. In this context, a given stimulus in one area can trigger significant consequences in others, which are not always fully anticipated due to the dynamism and unpredictability of the interactions that permeate such a system.

Complexity in an STS can be described and measured through specific attributes, such as a large number of dynamically interacting elements, high diversity of elements, unexpected variability, and resilience^(5)^. While the large number of dynamically interacting elements and the diversity of elements primarily refer to the properties that form the system, variability and resilience relate to functionality, reflecting the interactions that give rise to emergent phenomena.

The nursing professional practice environment and complex STS have been subjects of investigation both nationally and internationally. However, these constructs have not yet been explored together in the literature. Regarding the former, there are scientific studies in Latin American and European settings focused on themes such as safety attitudes and climate, care indicators, professional satisfaction, and burnout syndrome^(6-10)^. Complexity, on the other hand, is addressed in studies on healthcare services^(11)^, manufacturing systems^(12)^, and civil construction^(13)^.

Thus, understanding the relationship between the constructs of the professional practice environment and hospital sociotechnical complexity is relevant, as it will enable a better understanding of nursing work in a dynamic, diverse, and unpredictable setting, allowing for actions that promote more effective outcomes for quality and safety of care from the perspective of building a favorable environment. Based on the above, the following research question arises: What is the relationship between the nursing professional practice environment and hospital sociotechnical complexity?

## OBJECTIVES

To analyze the relationship between the nursing professional practice environment and hospital sociotechnical complexity from the perspective of nurses.

## METHODS

### Ethical Aspects

The study adhered to the ethical guidelines of Resolution 466/2012 of the National Health Council and was approved by the Research Ethics Committee of the proposing institution. At all planned stages, the Informed Consent Form was administered, ensuring that participants received information about the research. The authors of the instruments used for data collection were consulted to obtain permission for their use in this study. To ensure anonymity, each participant was assigned a letter based on their workplace (L = clinical inpatient unit; C = surgical inpatient unit; E = emergency; T = intensive care), followed by a numerical digit in ascending order of participation. In the interviews, the letter E was used, following the same numbering logic.

### Study Design, Period, and Location

This was a mixed-methods study with a sequential explanatory design, consisting of an initial quantitative phase (QUAN), which carried more weight and was cross-sectional, followed by a subsequent qualitative phase (qual), which was exploratory-descriptive^(14)^. Adopting the mixed-methods approach allowed for a more comprehensive and in-depth response to the research question by combining quantitative results from the instruments that classified the nursing practice environment and assessed hospital complexity with qualitative data that depicted the nurses’ lived experiences in the study setting. The presentation of findings and the writing of this manuscript followed the guidelines of the Mixed Methods Appraisal Tool^(15)^.

The quantitative and qualitative phases took place from November 2018 to April 2019 and from September to December 2019, respectively. The research was conducted in clinical and surgical inpatient units, emergency, and adult intensive care units at a large public university hospital (836 beds) located in southern Brazil, which has quality and safety standards certified by the Joint Commission International. At the time, the selected care areas comprised a total of 497 (59.4%) beds in the institution, of which 218 were clinical, 190 surgical, 50 in emergency, and 39 in intensive care.

### Population and Sample: Inclusion and Exclusion Criteria

In the quantitative phase, the sample included nurses providing care to adult patients in clinical inpatient units (n=72), surgical inpatient units (n=63), emergency (n=36), and intensive care units (n=55), during both day and night shifts, totaling 226 professionals. The sample size was calculated based on the recommendation of at least five respondents per item of the data collection instruments^(16)^, considering a 95% confidence level, a margin of error of two points, and an additional 10% to account for possible losses and refusals, resulting in a sample size of 132 nurses. The eligibility criteria for this phase were: employment duration of at least 90 days, a permanent employment contract, and being on active duty during the data collection period. Participants were selected through simple random sampling, using stratified drawing to ensure the maximum estimated number and proportional distribution in each area. In addition to the principal researcher, two trained research assistants approached the selected nurses in person during their work shifts to invite them to participate in the study. There were six refusals, and these participants were replaced, resulting in a final count of 42 professionals from clinical inpatient units, 37 from surgical units, 23 from emergency, and 30 from intensive care.

For the qualitative phase, all nurses who participated in the first phase were considered eligible, along with those interested in discussing the topic further. The principal researcher sent an invitation to participate via email, receiving favorable responses from seven professionals from clinical inpatient units, seven from surgical units, four from intensive care, and none from emergency. Given this scenario, other strategies were adopted: in the emergency department, nurses who returned the quantitative phase questionnaires close to the 48-hour deadline were invited on a case-by-case basis, with at least one participant selected from each work shift. In intensive care, the Snowball Technique was used, where the first professional who expressed interest in participating was asked to refer another colleague, and so on.

After these interventions, the total sample consisted of 20 nurses: 10 from clinical and surgical inpatient units, four from emergency, and six from intensive care. There were two withdrawals-one from emergency and one from intensive care-resulting in a final total of 18 nurses: five from clinical, five from surgical, five from intensive care units, and three from emergency, which met the criterion for data saturation^(17)^. In both phases, professionals hired on a temporary basis or those who were on leave for any reason during the data collection period were excluded.

### Data Collection and Organization

In the quantitative phase, the principal researcher and two research assistants administered the Brazilian version of the Practice Environment Scale-Nursing Work Index (PES-NWI) and the Complexity Characterization Questionnaire in person, along with a demographic and professional characterization form. This form included information on the department, shift, gender, age, type of education, years of experience in the profession and at the current workplace, employment status, and the number of patients attended to during the last shift.

The Brazilian version of the PES-NWI classifies the professional practice environment based on the presence of characteristics that facilitate nurses’ work. The scale covers 24 items distributed across five subscales: Nurse Participation in Hospital Affairs; Nursing Foundations for Quality of Care; Nurse Manager Ability, Leadership, and Support of Nurses/Nursing Staff; Staffing and Resource Adequacy; and Collegial Nurse-Physician Relations. It is a Likert-type scale ranging from one to four points (1 - Strongly Disagree; 2 - Disagree; 3 - Agree; 4 - Strongly Agree). Scores are obtained by averaging the responses, with a cutoff point of 2.5. Scores above this value in none or one subscale indicate unfavorable environments, in two or three subscales indicate mixed environments, and in four or five subscales indicate favorable environments^(18)^. Reliability was calculated using McDonald’s omega coefficient, with values above 0.82 for all subscales.

The Complexity Characterization Questionnaire assesses complexity in STS by measuring specific elements. The questionnaire consists of 23 questions across the four previously mentioned complexity attributes: Large Number of Dynamically Interacting Elements; High Diversity of Elements; Unexpected Variability; Resilience. The instrument features a continuous, increasing 15-centimeter line with endpoints labeled “Strongly Disagree” (1) and “Strongly Agree”^(15)^, where a higher degree of agreement indicates a greater presence of the attribute and its characteristics in the setting. Reliability was measured using McDonald’s omega coefficient, with a range from 0.47 to 0.78 among the attributes. In the original study^(19)^, conducted in an emergency service in the United States, low reliability of the scale was also identified, as indicated by Cronbach’s alpha calculation.

Preliminary analysis of the quantitative results supported the development of the semi-structured interview guide for the qualitative phase. The main topics addressed and explored in depth by the principal researcher with the participants included their understanding, based on their ideas and experiences, of the nursing professional practice environment and care complexity, the presence of complexity attributes in daily practice, and aspects related to the theme that influence the quality of care and patient safety. The interviews were conducted in person, scheduled according to the interviewee’s availability, and held in a private area within their work department. The interviews lasted an average of 29 minutes (SD = 9.6), were audio-recorded, and fully transcribed, with subsequent content validation by the participants. During the interviews, the repetition of information was observed, leading to saturation of the findings among the last participants, making further exploration of new elements unnecessary, as the proposed objectives had been achieved and the topic was explored in depth.

### Data Analysis

Initially, the quantitative data were organized into a Microsoft Excel spreadsheet, prepared by the principal researcher, with the assistance of a trained research assistant for double data entry to avoid inconsistencies. Subsequently, the Statistical Package for the Social Sciences (SPSS) version 18.0 was used to analyze continuous variables, which were presented as measures of central tendency (mean) and dispersion (standard deviation) or median and interquartile range. The normality of the sample distribution was verified using the Shapiro-Wilks test, while the existence and degree of association between variables were assessed using Spearman’s correlation coefficient and One-Way Analysis of Variance (ANOVA) with Tukey’s test. All inferences considered a 95% confidence interval (p ≤ 0.05), and the results for clinical and surgical inpatient units were grouped, considering common contextual characteristics.

For the analysis of qualitative data, NVivo version 11.0 software was used to organize the narratives from the semi-structured interviews in a structured manner. After literal transcription, the principal researcher subjected the narratives to thematic content analysis^(17)^, following the stages of pre-analysis, material exploration or coding, and processing/interpretation of the results obtained. This process resulted in three categories based on recurring themes: “An Environment Where Human and Technical Aspects Complement Each Other”, “Nurses’ Perspectives on Complexity”, and “Quality of Care and Patient Safety in a Complex Environment”.

**Figure 1:**
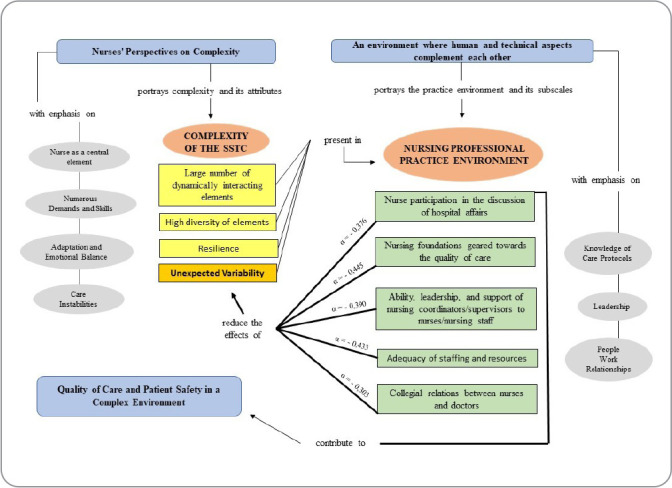
Integration of quantitative and qualitative results, Porto Alegre, Rio Grande do Sul, Brazil, 2021

Based on the premises of mixed-methods approaches, an integrated data analysis was conducted by connection^(14)^, using a conceptual map (Figure 1) to illustrate the quantitative and qualitative results. This approach aimed to explain and provide new insights into how the constructs of the professional practice environment and hospital sociotechnical complexity are related, seeking convergences, divergences, and/or complementarity among the findings.

## RESULTS

The demographic and professional characterization in the quantitative phase showed a predominance of female participants, with 116 (87.9%) of the sample, and an average age of 41.6 years (SD = 8.2). The median time since graduation was 13 years (10.5-21.0), with a prevalence of specialist degrees held by 88 participants (66.7%). Most of the sample maintained only one employment relationship, 112 (84.8%), and had been employed at the hospital for a median of 8.1 years (0.8-38.5), of which 6.5 years (4.0-11.0) were spent in their current work departments. The night shift stood out as the primary shift for these professionals, with 55 (41.7%) working during this time. The number of patients assigned per nurse during the last shift showed a median of 17.5 (13.7-21.0) in clinical-surgical inpatient units, 50.0 (23.0-58.0) in emergency, and 5.0 (5.0-5.0) in intensive care.

In the qualitative phase, the participants were also predominantly female, with ages ranging from 32 to 53 years, and most worked the day shift. The professional practice environment was classified as favorable in all study areas, based on the application of the Brazilian version of the PES-NWI (Table 1).

**Table 1:** Classification of the Professional Practice Environment Based on the Application of the Brazilian Version of the PES-NWI, Porto Alegre, Rio Grande do Sul, Brazil, 2021

Subscales	Clinical-Surgical Inpatient Units (n=79)	Emergency (n=23)	Intensive Care (n=30)	*p* value
Nurse Participation in Hospital Affairs	2.96 + 0.59)	2.53 + 0.55^^*^^	3.27 + 0.54^^*^^	0.000^^**^^
Nursing Foundations for Quality of Care	3.13 + 0.48^^*^^	2.57 + 0.46^^*^^	3.54 + 0.36^^*^^	0.000^^**^^
Nurse Manager Ability. Leadership. and Support of Nurses/Nursing Staff	3.23 + 0.57^^*^^	2.75 + 0.62^^*^^	3.18 + 0.57	0.003
Staffing and Resource Adequacy	2.62 + 0.60^^*^^	1.88 + 0.61^^*^^	3.23 + 0.61^^*^^	0.000^^**^^
Collegial Nurse-Physician Relations	2.90 + 0.58	2.89 + 0.55	2.82 + 0.59	0.787

* ANOVA One-Way with Tukey’s test;

** p ≤ 0,05.

Such perception was reinforced by the participants’ narratives in the category “An Environment Where Human and Technical Aspects Complement Each Other”, primarily due to the existence of protocols that ensure good practices and improve the quality of care, leading to advances in knowledge and satisfaction in working at the hospital.

*We have good working conditions, which allow us to pursue knowledge. We have qualified colleagues.* (L31-E11)

*So, when I am asked if anything is lacking here in the hospital for patient or worker safety, I say that this is the most developed place I have ever worked, because there is a concern and an effort to ensure that* [patient safety] *is present every day, that it is worked on every day. Regarding this aspect, I believe our institution is quite advanced.* (C36-E14)

It is noteworthy in Table 1 that the subscale “Staffing and Resource Adequacy” showed a score of 1.88 (SD = 0.61) in the emergency department, below the cutoff point, indicating an unfavorable perception of the environment compared to clinical-surgical inpatient and intensive care units (p=0.000), likely due to the high number of patients assigned per shift. Concerns about staffing levels were evident in the emergency nurses’ discourse in the category “Quality of Care and Patient Safety in a Complex Environment”, corroborating the numerical findings. The nurses expressed that the demand for walk-in care tends to cause overcrowding, and in this situation, inadequate staffing exposes patients to risks and care failures.

[...] *because if you have the appropriate number of patients, you can provide adequate care* [...]? (E22-E15)

[...] *the overcrowding here in the emergency department is one of the factors that greatly impacts our care practice, our care for patients, the risks* [...] *there are more risks of medication errors, patient mix-ups, the care we should provide, even basic care, sometimes we can’t manage.* (E9-E16)

Regarding the complexity attributes in the professional practice environment, the application of the Complexity Characterization Questionnaire showed that these attributes were recognized by the nurses (Table 2).

**Table 2:** Presence of complexity attributes in the professional practice environment based on the complexity characterization questionnaire, Porto Alegre, Rio Grande do Sul, Brazil, 2021

Complexity Attributes	Clinical-Surgical Inpatient Units (n=79)	Emergency (n=23)	Intensive Care (n=30)	*p* value
Large Number of Dynamically Interacting Elements	10.99 + 2.07	11.02 + 1.59	12.21 + 1.72	0.011
High Diversity of Elements	11.12 + 3.08	11.34 + 2.62	12.28 + 2.42	0.173
High Diversity of Elements	5.61 + 1.71^^*^^	6.90 + 2.16^^*^^	5.38 + 1.70^^*^^	0.005^^**^^
Resilience	9.71 + 1.85	9.80 + 1.39	9.90 + 1.60	0.863

* ANOVA One-Way with Tukey’s test;

** p ≤ 0,05.

In the interviews, examples of professional experiences in the category “Nurses’ Perspectives on Complexity” confirmed these results. The “Large Number of Dynamically Interacting Elements” was related to the nurse being a central figure who navigates among various areas and teams. The “High Diversity of Elements” was associated with the intense demand for activities, and “Resilience” was linked to adaptive capacity in the face of adversity, as illustrated below:

[...] *the nurse, being a central element of care, has to interact with all professional categories, manage their own team, and manage patient care.* [...] *They have to do all this simultaneously so that work happens in a synchronized way.* (L4-E9)

[...] *it’s family members asking questions, it’s the patient wanting to know the test results,* [...] *whether they’ll be discharged.* [...] *I have to keep up. Besides the procedures* [...]*. We have a significant workload.* (E22-E15)

[...] *resilience comes from our ability* [...] *to know how to reorganize and restructure ourselves.* (T23-E10)

Regarding “Unexpected Variability”, scores below 7 and close to “strongly disagree” in Table 2 tend to indicate that nurses did not recognize, or barely recognized, the presence of characteristics linked to this attribute. When comparing areas (p=0.005), the emergency department showed a higher mean for this element, which may be justified by the lack of control in the sector, as the teams incorporate the variability of demand as part of the process. Statements in the category “Nurses’ Perspectives on Complexity” acknowledged this attribute as an inherent trait of the care routine while also bringing up aspects related to work methods in different areas.

[...] *sometimes things can be very calm and stable with the patient, the team, and everything, but then suddenly everything changes* [...]. (T21-E13)

*Unexpected variability, we don’t have so much unexpected variability, in terms of patients, because it’s a more controlled environment* [intensive care]*, unlike the emergency department, you know you have five beds, five patients* [...]. (T17-E5)

In contrast, when associating the subscales of the Brazilian version of the PES-NWI with the complexity attributes (Table 3), “Unexpected Variability” was the only attribute that showed a significant (p<0.01) moderate and inverse correlation, suggesting that it can be reduced through certain characteristics present in the practice environment.

**Table 3:** Correlation between the subscales of the Brazilian version of the PES-NWI and the attributes of the Complexity Characterization Questionnaire, Porto Alegre, Rio Grande do Sul, Brazil, 2021

PES-NWI Brazilian Version Subscales	Complexity Characterization Questionnaire Attributes			
	Large number of dynamically interacting elements	High diversity of elements	Unexpected variability	Resilience
Nurse Participation in Hospital Affairs	0.126	-0.025	-0.376^^*^^	0.100
Nursing Foundations for Quality of Care	0.120	-0.031	-0.445^^*^^	0.061
Nurse Manager Ability. Leadership. and Support of Nurses/Nursing Staff	-0.031	-0.188	-0.390^^*^^	-0.001
Staffing and Resource Adequacy	0.039	-0.094	-0.433^^*^^	0.120
Collegial Nurse-Physician Relations	0.010	-0.110	-0.303^^*^^	0.159

ǂ ANOVA One-Way with Tukey’s test;

* P < 0,01.

Regarding this finding, in the category “An Environment Where Human and Technical Aspects Complement Each Other,” it was mentioned that a professional practice environment with elements favorable to nurses’ activities tends to minimize traits of “Unexpected Variability” and contributes to the quality of care and patient safety.

[...] *a team that comes to work cohesive, happy, feeling valued, is a team that will provide higher quality care for my patients, and if, in addition, I ensure they have adequate training, the right materials to work with, that their rights are respected, and they have their time off* [...] *we can provide quality and safety for the patient.* (L4-E9)

The conceptual map (Figure 1) represents the integration of quantitative and qualitative results, with the central elements being the constructs “nursing professional practice environment” and “sociotechnical system complexity” - both highlighted in red, with subscales (green) and attributes (yellow), respectively - connected with the three categories (blue) and subthemes that emerged from the semi-structured interviews.

This revised translation ensures clarity and maintains technical accuracy, making it suitable for a scientific and professional audience.

## DISCUSSION

The professional practice environment was perceived as favorable by the nurses, as the subscales of the Brazilian version of the PES-NWI showed four to five domains above 2.5 in all areas. Interviews with participants reinforced this result, indicating that the hospital’s certification and protocols directed toward care practices based on quality and patient safety tend to influence job satisfaction and a positive perception of their own work^(20)^. However, this finding differs from recent studies in other Brazilian settings that used the same measurement instrument^(21-23)^.

On the other hand, the evaluation of the “Staffing and Resource Adequacy” subscale of the Brazilian version of the PES-NWI was unfavorable in the emergency department when compared to clinical-surgical and intensive care units, possibly due to the disproportionate ratio of patients assigned per shift versus the number of professionals. Adequate staffing was mentioned by participants in this area as a fundamental aspect, understood to influence the quality of care and mitigate risks that compromise nursing care.

The issue of human resource provision is a constant theme in nursing research, mainly because it is considered an element that directly affects the quality of care and the safety of assistance^(21,24)^, a finding similar to the present study. Thus, the “Staffing and Resource Adequacy” subscale commonly shows the lowest scores when evaluating the practice environment^(6,20,25)^, a trend also observed, although only in the emergency department. A number of patients exceeding the estimated capacity, in addition to creating work overload, increases the likelihood of patient safety incidents, arising from the need for adjustments and improvised solutions due to the excessive processes that need to be managed by the teams^(26)^. The institution in this study encourages the reporting of incidents and adverse events, allowing errors to be seen systemically and as opportunities for improvement, demystifying the punitive culture that still persists in many institutions. It is necessary to continuously rethink this process, as it provides relevant data for analyses and evaluations, implementation of barriers, and reviews of care and management flows.

The application of the Complexity Characterization Questionnaire indicated that nurses in clinical-surgical inpatient units, emergency departments, and intensive care units recognized the presence of characteristics that constitute the complexity attributes “Large Number of Dynamically Interacting Elements”, “High Diversity of Elements”, and “Resilience” within their practice environment. This recognition reveals that nursing work in the hospital sociotechnical system is complex. During the interviews, participants provided examples from their daily practical situations that demonstrated the presence of these attributes in their environment, reinforcing this understanding. Thus, the degree of patient dependency, the number of demands, and the need to encompass multiple skills and knowledge for efficient work support the assertion that the healthcare sector is a complex system^(2)^. A study conducted in clinical-surgical inpatient units^(27)^ showed that nurses feel challenged when working in high-pressure settings, striving to turn difficulties into learning opportunities, even as they work to change such realities.

Conversely, “Unexpected Variability” showed lower scores compared to the other attributes, and in the nurses’ narratives, it was associated with fluctuations in patients’ health conditions, indicating that remnants of uncertainty and unpredictability are embedded in their daily work routines. Regarding the higher score for this item in the emergency department, it is possible that walk-in care, or the so-called open-door policy, influenced the quantitative result, as such an approach makes it difficult for teams to anticipate and control events. In this context, clinical-surgical inpatient units and intensive care units present a more controlled environment and, consequently, experience fewer variations.

When correlating the Brazilian version of the PES-NWI with the Complexity Characterization Questionnaire, the results indicated that less “Unexpected Variability” leads to a better professional practice environment, even though this is an inherent characteristic of nursing work. This finding was evident in the interviews, as the nurses expressed that an environment with structural and relational elements favorable to work contributes to the quality of care and patient safety and is less likely to be influenced by the variations that permeate the system and pose care risks. This finding is consistent with recent studies that suggest an environment with established favorable characteristics is less likely to be influenced by the variations^(7,28,29)^.

### Study limitations

This research refers to a specific time frame prior to the Coronavirus pandemic, conducted in a single institution and with a sample restricted to nursing professionals. Additionally, the internal consistency of the Complexity Characterization Questionnaire was low, which may be attributed to its limited dissemination and application experience compared to the PES-NWI, indicating a need for future research to provide more conclusive evidence about its reliability.

### Contributions to the field of Nursing

This study introduces reflections on the complexity of nursing work and the elements that foster a practice environment favorable to care, aligning with current sociopolitical movements advocating for the professional recognition of the nursing profession. It also legitimizes the recommendations of the World Health Organization and nursing representative entities, which encourage the production of scientific evidence related to work conditions, quality of care, and patient safety in practice settings.

## CONCLUSIONS

The study results indicated a relationship between the nursing professional practice environment and sociotechnical complexity in the hospital context, allowing for an understanding of the characteristics of the areas studied and their implications for the quality of care and safety of work processes. Complexity was shown to be embedded in the practice environment, which, when presenting favorable characteristics, tends to minimize the inherent variability of daily routines and, consequently, maintain greater system stability. The importance of conducting research to identify favorable points and/or weaknesses in practice environments is also highlighted, as the results will provide opportunities to advance strategies focused on improving patient care and professional satisfaction.

## Data Availability

https://doi.org/10.48331/scielodata.LYJ9X6

## References

[1] Braithwaite J, Glasziou P, Westbrook J. (2020). The three numbers you need to know about healthcare: the 60-30-10 challenge. BMC Med.

[2] Carthon JMB, Davis L, Dierkes A, Hatfield L, Hedgeland T, Holland S (2019). Association of Nurse Engagement and Nurse Staffing on Patient Safety. J Nurs Care Qual.

[3] Churruca K, Pomare C, Ellis LA, Long JC, Braithwaite J. (2019). The influence of complexity: a bibliometric analysis of complexity science in healthcare. BMJ Open.

[4] McNab D, McKay J, Shorrok S, Luty S, Bowie P. (2020). Development and application of ‘systems thinking’ principles for quality improvement. BMJ Open Quality.

[5] Saurin TA, Gonzalez SS. (2013). Assessing the compatibility of the management of standardized procedures with the complexity of a sociotechnical system: case study of a control room in an oil refinery. App Ergonom.

[6] Riboldi CO, Gasparino RC, Kreling A, Oliveira NJ, Barbosa AS, Magalhães AMM. (2021). Environment of the professional nursing practice in Latin American countries: a scoping review. Online Braz J Nurs.

[7] Lake ET, Sanders J, Duan R, Riman KA, Schoenauer KM, Chen Y. A (2019). Meta-Analysis of the associations between the nurse work environment in hospitals and 4 sets of outcomes. Med Care.

[8] Neves TMA, Parreira PMSD, Rodrigues VJL, Graveto JMGN. (2012). Impact of safe nurse staffing on the quality of care in Portuguese public hospitals: a cross-sectional study. J Nurs Manag.

[9] Brešan M, Erčulj C, Lajovic J, Ravljen M, Sermeus W, Grosek S. (2021). The relationship between the nurses’ work environment and the quality and safe nursing care: Slovenian study using the RN4CAST questionnaire. PLoS One.

[10] Viscardi MK, French R, Brom H, Lake E, Ulrich C, McHugh MD. (2022). Care quality, patient safety, and nurse outcomes at hospitals serving economically disadvantaged patients: a case for investment in nursing. Policy Polit Nurs Pract.

[11] Saurin TA, Wachs P, Bueno WP, Kuchenbecker RS, Boniatti MM, Zani CM (2022). Coping with complexity in the COVID pandemic: an exploratory study of intensive care units. Hum Factors Ergons Manuf.

[12] Soliman M, Saurin TA, Anzanello MJ. (2018). The impacts of lean production on the complexity of socio-technical systems. Int J Produc Econom.

[13] Peñaloza GA, Saurin TA, Formoso CT. (2020). Monitoring complexity and resilience in construction projects: the contribution of safety performance measurement systems. App Ergonom.

[14] Harrison RL, Reilly TM, Creswell JW. (2020). Methodological rigor in mixed methods: an application in management studies. J Mixed Methods Res.

[15] (2018). Mixed Methods Appraisal Tool (MMAT) Version 2018.

[16] Hair JF, Black WC, Babin BJ (2009). Análise multivariada de dados.

[17] Minayo MCS. (2017). Amostragem e saturação em pesquisa qualitativa: consensus e controvérsias. Rev Pesqui Qual.

[18] Gasparino RC, Guirardello EB. (2017). Validation of the Practice Environment Scale to the Brazilian culture. J Nurs Manag.

[19] Righi AW, Wachs P, Saurin TA., Arezes PM (2019). Proposal of an instrument for the characterization of complex socio-technical systems: a study of an emergency department. Occupational and Environmental Safety and Health.

[20] Oliveira JLC, Magalhães AMM, Bernardes A, Haddad MCFL, Wolf LDG, Marcon SS (2019). Influence of hospital accreditation on professional satisfaction of the nursing team: mixed method study. Rev Latino-Am Enfermagem.

[21] Gasparino RC, Ferreira TDM, Carvalho KMA, Rodrigues ESA, Tondo JCA, Silva VA. (2019). Evaluation of the professional practice environment of nursing in health institutions. Acta Paul Enferm.

[22] Yanarico DMI, Balsanelli AP, Gasparino RC, Bohomol E. (2020). Classification and evaluation of environment of the professional nursing practice in a teaching hospital. Rev Latino-Am Enfermagem.

[23] Maciel KRL, Ribeiro AC, Souza MRC. (2022). Análise das características intervenientes do ambiente de prática do enfermeiro no espaço do hospital. Res, Soc Develop.

[24] Gasparino RC, Ferreira TDM, Oliveira HC, Alves DFS, Balsanelli AP. (2021). Leadership, adequate staffing and material resources, and collegial nurse: physician relationships promote better patients, professionals and institutions outcomes. J Adv Nurs.

[25] Ambani Z, Kutney-Lee A, Lake ET. (2020). The nursing practice environment and nurse job outcomes: a path analysis of survey data. J Clin Nurs.

[26] Anderson JE, Ross AJ, Back J, Duncan M, Jaye P., Wiigs S, Fahlbruch B (2019). Resilience engineering as a quality improvement method in healthcare. Exploring Resilience: a scientific journey from practice to theory.

[27] Van Bogaert P, Peremans L, Van Heusden D, Verspuy M, Kureckova V, Van de Cruys Z (2017). Predictors of burnout, work engagement and nurse reported job outcomes and quality of care: a mixed method study. BMC Nurs.

[28] Gensimore MM, Maduro RS, Morgan MK, McGee GW, Zimbro KS. (2020). The effect of nurse practice environment on retention and quality of care via burnout, work characteristics, and resilience: a moderated mediation model. J Nurs Adm.

[29] Nascimento A, Jesus É. (2020). Nursing Work Environment and patient outcomes in a hospital context: a scoping review. J Nurs Adm.

