# Supplementary material for: The nursing practice environment and hospital sociotechnical complexity: a mixed-methods study
Source: Rev Bras Enferm. 2024 Dec 16;77(6):e20230315. doi: 10.1590/0034-7167-2023-0315 (PMC11654510; doi:10.1590/0034-7167-2023-0315)
Supplement: Translation

# Translation: O ambiente de prática da enfermagem e a complexidade sociotécnica hospitalar: estudo de método misto

**DOI:** 10.1590/0034-7167-2023-0315pt

**Authors:** Caren de Oliveira Riboldi, Renata Cristina Gasparino, Wiliam Wegner, Eder Henriqson, Tarcísio Abreu Saurin, Ana Maria Müller de Magalhães

**Affiliations:** I Hospital de Clínicas de Porto Alegre. Porto Alegre, Rio Grande do Sul, Brasil; II Universidade Estadual de Campinas. Campinas, São Paulo, Brasil; III Universidade Federal do Rio Grande do Sul. Porto Alegre, Rio Grande do Sul, Brasil; IV Pontifícia Universidade Católica do Rio Grande do Sul. Porto Alegre, Rio Grande do Sul, Brasil

**Keywords:** Análise da Complexidade, Ambiente de Instituições de Saúde, Enfermagem, Segurança do Paciente, Qualidade da Assistência à Saúde

## Abstract

**Objetivos:**

analisar a relação entre o ambiente de prática profissional da enfermagem e a complexidade sociotécnica hospitalar na percepção de enfermeiros.

**Métodos:**

estudo explanatório sequencial de métodos mistos realizado em hospital do sul do Brasil. Aplicou-se a versão brasileira da *Practice Environment Scale-Nursing Work Index* e o Questionário de Caracterização da Complexidade para 132 enfermeiros. Após, realizaram-se entrevistas semiestruturadas com 18 participantes, submetidas à análise temática. A integração dos dados foi por conexão.

**Resultados:**

o ambiente de prática revelou-se favorável, exceto na subescala Adequação da equipe e de recursos, e a complexidade presente nas atividades. As três categorias emergentes explicaram aspectos humanos e técnicos, relacionados com a complexidade no ambiente de prática, qualidade do cuidado e segurança do paciente. A variabilidade inesperada correlacionou-se inversamente com o ambiente de prática.

**Conclusões:**

os resultados do estudo apontam relação entre os construtos, com implicações para a qualidade e segurança do cuidado.

## INTRODUÇÃO

As organizações hospitalares de grande porte e com altos níveis de exigência assistencial possuem peculiaridades que tendem a amplificar sua complexidade, tais como diversidade de patologias e especialidades profissionais, pacientes com múltiplas doenças crônicas e gravidade clínica, demanda por serviços de difícil substituição e o uso de tecnologias diagnósticas e terapêuticas inovadoras, entre outras. Além disso, todos esses elementos não se comportam de maneira isolada ou linear, visto que estão submetidos aos estímulos, recursos, informações e feedbacks presentes no ambiente onde estão incorporados^(1)^.

Na enfermagem, o local onde as ações de cuidado ocorrem e tais elementos interagem é denominado ambiente de prática profissional, sendo definido pela presença ou ausência de características que facilitam ou restringem o desenvolvimento das atividades. Entre os aspectos que tornam um ambiente favorável ao trabalho estão a autonomia profissional, tomada de decisão descentralizada, cuidados clínicos de alta qualidade, liderança participativa, oportunidades de qualificação e progressão de carreira, e relações colegiadas colaborativas^(2)^.

Os serviços de saúde são considerados sistemas sociotécnicos (SST) de alta complexidade, compostos por elementos sociais (pessoas e suas relações) e técnicos (por exemplo, equipamentos e *software*) interdependentes^(3,4)^. Neste cenário, um dado estímulo em uma área pode desencadear consequências importantes nas demais, nem sempre totalmente antecipadas devido ao dinamismo e à imprevisibilidade das interações que permeiam tal sistema.

A complexidade em um SST pode ser descrita e mensurada a partir de atributos específicos, tais como o grande número de elementos que interagem dinamicamente, grande diversidade de elementos, variabilidade inesperada e resiliência^(5)^. Enquanto o grande número de elementos que interagem dinamicamente e a diversidade de elementos referem-se, principalmente, às propriedades que formam o sistema, a variabilidade e a resiliência relacionam-se com a funcionalidade, refletindo as interações que originam fenômenos emergentes.

O ambiente de prática profissional da enfermagem e os SST complexos têm sido objeto de investigação no âmbito nacional e internacional. No entanto, tais construtos ainda não foram explorados na literatura de forma conjunta. Quanto ao primeiro, há produções científicas no cenário latino-americano e europeu voltadas para temas como atitudes e clima de segurança, indicadores assistenciais, satisfação profissional e síndrome de Burnout^(6-10)^. Já a complexidade é abordada em estudos nos serviços de saúde^(11)^, sistemas de manufatura^(12)^ e construção civil^(13)^.

Assim, pondera-se que conhecer a relação entre os construtos ambiente de prática profissional e complexidade sociotécnica hospitalar é relevante, na medida em que possibilitará compreender o trabalho de enfermagem em um cenário dinâmico, diverso e imprevisível, oportunizando ações que promovam resultados mais eficazes para a qualidade e segurança do cuidado na perspectiva da construção de um ambiente favorável. Com base no exposto, remete-se à seguinte questão de pesquisa: qual a relação entre o ambiente de prática profissional da enfermagem e a complexidade sociotécnica hospitalar?

## OBJETIVOS

Analisar a relação entre o ambiente de prática profissional da enfermagem e a complexidade sociotécnica hospitalar na percepção de enfermeiros.

## MÉTODOS

### Aspectos éticos

O estudo atendeu às recomendações éticas da Resolução 466/2012 do Conselho Nacional de Saúde e foi aprovado pelo Comitê de Ética em Pesquisa da instituição proponente. Em todas as etapas previstas, foi aplicado o Termo de Consentimento Livre e Esclarecido, assegurando aos participantes a obtenção de informações sobre a pesquisa. Os autores dos instrumentos aplicados na coleta de dados foram consultados para anuência de utilização nesta proposta. A fim de garantir o anonimato, cada participante recebeu uma letra de acordo com o seu local de trabalho (L = unidade de internação clínica; C = unidade de internação cirúrgica; E = emergência; T = terapia intensiva), seguida de um algarismo numérico em ordem crescente de participação. Nas entrevistas, optou-se pela letra E, adotando-se a mesma lógica de numeração.

### Desenho, período e local do estudo

Estudo de métodos mistos com desenho explanatório sequencial, composto por uma etapa inicial quantitativa (QUAN), com maior peso e do tipo transversal, e outra subsequente qualitativa (qual), de caráter exploratório-descritivo^(14)^. A adoção da abordagem mista permite responder à questão de pesquisa de forma mais abrangente e aprofundada, combinando os resultados quantitativos provenientes dos instrumentos de classificação do ambiente de prática da enfermagem e de avaliação da complexidade hospitalar, com os dados qualitativos que retrataram a experiência vivenciada pelos enfermeiros no cenário em estudo. A apresentação dos achados e a redação deste manuscrito seguiram as diretrizes do *Mixed Methods Appraisal Tool* ^(15)^.

As etapas quantitativa e qualitativa ocorreram entre novembro de 2018 e abril de 2019, e de setembro a dezembro de 2019, respectivamente. A pesquisa foi conduzida em unidades de internação clínica e cirúrgica, emergência e terapia intensiva de pacientes adultos, em um hospital público, universitário e de grande porte (836 leitos), localizado na região sul do Brasil, o qual apresenta padrões de qualidade e segurança certificados pela *Joint Commission International*. Na ocasião, as áreas assistenciais elencadas totalizavam 497 (59,4%) leitos na instituição, dos quais 218 clínicos, 190 cirúrgicos, 50 na emergência e 39 de terapia intensiva.

### População e amostra: critérios de inclusão e exclusão

Na etapa quantitativa, a amostra contemplou os enfermeiros que atuavam na assistência de pacientes adultos em unidades de internação clínica (n=72), cirúrgica (n=63), emergência (n=36) e terapia intensiva (n=55), nos turnos diurno e noturno, totalizando 226 profissionais. O cálculo da amostra baseou-se na recomendação de ao menos cinco respondentes por item dos instrumentos de coleta de dados^(16)^, considerando um nível de confiança de 95%, margem de erro de dois pontos e o acréscimo de 10% para eventuais perdas e recusas, resultando no tamanho amostral de 132 enfermeiros. Os critérios de elegibilidade nesta fase foram: vínculo empregatício igual ou superior a 90 dias, contratação definitiva e estar em atividade laboral durante o período da coleta de dados. A seleção dos participantes ocorreu através de amostragem aleatória simples, mediante sorteio por estratos, garantindo assim o número máximo estimado e a distribuição proporcional em cada área. Além da pesquisadora principal, duas pesquisadoras auxiliares capacitadas abordaram presencialmente os enfermeiros selecionados, em seus turnos de trabalho, realizando o convite de participação na pesquisa. Houve seis negativas, substituindo-se esses participantes e contabilizando, ao final, 42 profissionais de unidades de internação clínica, 37 da cirúrgica, 23 da emergência e 30 da terapia intensiva.

Para a etapa qualitativa, foram considerados elegíveis todos os enfermeiros que participaram da primeira fase, somado ao desejo em discutir a temática. A pesquisadora principal encaminhou um convite de participação por e-mail, obtendo retorno favorável de sete profissionais das unidades de internação clínica, sete da cirúrgica, quatro da terapia intensiva e nenhuma manifestação da emergência. Diante deste panorama, adotaram-se outras estratégias: na emergência, optou-se por convidar, pontualmente, enfermeiros que efetuaram a devolução dos questionários da etapa quantitativa próximo às 48 horas estabelecidas, selecionando-se ao menos um participante em cada turno de trabalho; e na terapia intensiva, adotou-se a Técnica de *Snowball*, solicitando ao primeiro profissional que manifestou interesse em participar da pesquisa a indicação de outro colega entre seus pares, e assim sucessivamente.

Após essas intervenções, a amostra totalizou 20 enfermeiros, sendo 10 das unidades de internação clínica e cirúrgica, quatro da emergência e seis da terapia intensiva. Ainda, houve uma desistência na emergência e outra na terapia intensiva, perfazendo, ao final, 18 enfermeiros - cinco de unidades clínicas, cirúrgicas e terapia intensiva, cada uma, e três da emergência - o que permitiu atender ao critério de saturação das informações^(17)^. Em ambas as etapas, adotou-se a exclusão dos profissionais contratados na modalidade temporária ou afastados por qualquer motivo durante o período da coleta de dados.

### Coleta e organização dos dados

Na etapa quantitativa, a pesquisadora principal e as duas auxiliares de pesquisa aplicaram presencialmente os instrumentos *Practice Environment Scale-Nursing Work Index* (PES-NWI) versão brasileira e o Questionário de Caracterização da Complexidade, além de uma ficha de caracterização demográfica e profissional, que incluía setor, turno, sexo, idade, tipo de formação, tempo de experiência na profissão e no local atual de trabalho, vínculos empregatícios e número de pacientes atendidos no último plantão.

A PES-NWI versão brasileira classifica o ambiente de prática profissional com base na presença de características que favorecem o trabalho do enfermeiro. A escala abrange 24 itens distribuídos em cinco subescalas: Participação dos enfermeiros na discussão dos assuntos hospitalares; Fundamentos de enfermagem voltados para a qualidade do cuidado; Habilidade, liderança e suporte dos coordenadores/supervisores de enfermagem aos enfermeiros/equipe de enfermagem; Adequação da equipe e de recursos; e Relações colegiais entre enfermeiros e médicos. A escala é do tipo Likert e varia de um a quatro pontos (1 - Discordo totalmente; 2 - Discordo parcialmente; 3 - Concordo parcialmente; 4 - Concordo totalmente). Os escores são obtidos pela média das respostas e apresentam o ponto de corte 2,5, de modo que pontuações acima deste valor em nenhuma ou uma subescala referem-se a ambientes desfavoráveis, em duas ou três subescalas a ambientes mistos e em quatro ou cinco subescalas, a ambientes favoráveis^(18)^. A confiabilidade foi calculada por meio do coeficiente ômega de McDonald, obtendo-se valores acima de 0,82 em todas as subescalas.

O Questionário de Caracterização da Complexidade avalia a complexidade em SST a partir da mensuração de elementos específicos. O questionário contempla 23 questões dispostas entre os quatro atributos de complexidade previamente mencionados: Grande número de elementos que interagem dinamicamente; Grande diversidade de elementos; Variabilidade inesperada; Resiliência. O instrumento apresenta uma linha contínua e crescente de 15 centímetros com os extremos discordo totalmente (1) e concordo totalmente^(15)^, onde quanto maior o grau de concordância, maior a presença do atributo e suas características no local. A confiabilidade foi aferida por meio do coeficiente ômega de McDonald, com variação de 0,47 a 0,78 entre os atributos. No estudo original^(19)^, desenvolvido em um serviço de emergência nos Estados Unidos, também se identificou baixa confiabilidade da escala mediante cálculo do alfa de Cronbach.

A análise preliminar dos resultados quantitativos subsidiou a elaboração do roteiro para as entrevistas semiestruturadas previstas na etapa qualitativa. Os principais pontos abordados e aprofundados pela pesquisadora principal com os participantes exploraram o entendimento dos mesmos, a partir de suas ideias e experiências, sobre o ambiente de prática profissional da enfermagem e a complexidade assistencial, a presença dos atributos de complexidade no dia a dia e aspectos relacionados à temática que influenciam na qualidade do cuidado e segurança do paciente. As entrevistas ocorreram de forma presencial, agendadas conforme a disponibilidade do entrevistado e em local reservado no setor de trabalho, com duração média de 29 minutos (DP = 9,6), gravação em áudio e transcrição na íntegra, com posterior validação do conteúdo pelos profissionais. Durante o desenvolvimento das entrevistas, observou-se a repetição de informações, culminando na saturação dos achados entre os últimos participantes, não sendo necessária a busca por novos elementos, visto o alcance do objetivo proposto e a profundidade do tema explorado.

### Análise dos dados

Inicialmente, os dados quantitativos foram organizados em uma planilha do Microsoft Excel, elaborada pela pesquisadora principal, contando com um auxiliar de pesquisa capacitado para o procedimento de dupla digitação, a fim de evitar inconsistências nas informações. Posteriormente, utilizou-se o *Statistical Package for Social Science* (SPSS) versão 18.0 para análise das variáveis contínuas, apresentadas como medidas de tendência central (média) e dispersão (desvio padrão) ou mediana e intervalo interquartílico. A normalidade de distribuição da amostra foi verificada pelo teste de Shapiro-Wilks, enquanto a existência e o grau de associação entre variáveis foram apurados por meio do coeficiente de correlação de Spearman e Análise da Variância (ANOVA) *One-Way* com teste de Tukey. Em todas as inferências, considerou-se o intervalo de confiança de 95% (p ≤ 0,05), e os resultados referentes às unidades de internação clínica e cirúrgica foram agrupados, considerando-se características contextuais em comum.

Na análise dos dados qualitativos, utilizou-se o *software* NVivo versão 11.0 para organizar, de maneira estruturada, os relatos oriundos das entrevistas semiestruturadas. A pesquisadora principal submeteu os relatos à análise temática de conteúdo^(17)^ após transcrição literal, seguindo as etapas de pré-análise, exploração do material ou codificação e tratamento dos resultados obtidos/interpretação, o que originou três categorias com base nos temas recorrentes: Um ambiente onde o humano e o técnico se complementam; O olhar dos enfermeiros para a complexidade; Qualidade do cuidado e segurança do paciente em um ambiente complexo.

A partir das premissas que compõem a vertente de métodos mistos, procedeu-se à análise integrada dos dados, por conexão^(14)^, utilizando-se um mapa conceitual (Figura 1) para ilustrar os resultados quantitativos e qualitativos, com vistas a explicar e estabelecer novos insights sobre como os construtos ambiente de prática profissional e complexidade sociotécnica hospitalar se relacionam, buscando convergências, divergências e/ou a complementaridade entre os achados.

**Figura 1:** Integração dos resultados quantitativos e qualitativos, Porto Alegre, Rio Grande do Sul, Brasil, 2021

## RESULTADOS

A caracterização demográfica e profissional, na etapa quantitativa, demonstrou predomínio do sexo feminino, com 116 (87,9%) participantes, e média de idade de 41,6 anos (dp = 8,2). O tempo de formação apresentou mediana de 13 anos (10,5-21,0), com prevalência de titulação de especialista, 88 (66,7%). A maior parte da amostra mantinha apenas um vínculo empregatício, 112 (84,8%), e estavam contratados no hospital há 8,1 anos (0,8-38,5), dos quais 6,5 anos (4,0-11,0) lotados nos setores atuais de trabalho. O turno noturno destacou-se como o principal turno de atuação dos profissionais, com 55 (41,7%). O número de pacientes atribuídos no último plantão por enfermeiro apresentou mediana de 17,5 (13,7-21,0) nas unidades de internação clínica-cirúrgica, 50,0 (23,0-58,0) na emergência e 5,0 (5,0-5,0) na terapia intensiva.

Na fase qualitativa, os participantes também se mostraram majoritariamente do sexo feminino, com idades variando de 32 a 53 anos, sendo a maioria com atuação laboral no turno diurno. O ambiente de prática profissional foi classificado como favorável em todas as áreas do estudo, com base na aplicação da PES-NWI versão brasileira (Tabela 1).

**Tabela 1:** Classificação do ambiente de prática profissional com base na aplicação da PES-NWI versão brasileira, Porto Alegre, Rio Grande do Sul, Brasil, 2021

Subescalas	Unidades de internação clínica-cirúrgica (n=79)	Emergência (n=23)	Terapia intensiva (n=30)	Valor de *p*
Participação dos enfermeiros na discussão dos assuntos hospitalares	2,96 + 0,59)	2,53 + 0,55^^*^^	3,27 + 0,54^^*^^	0,000^^**^^
Fundamentos de enfermagem voltados para a qualidade do cuidado	3,13 + 0,48^^*^^	2,57 + 0,46^^*^^	3,54 + 0,36^^*^^	0,000^^**^^
Habilidade, liderança e suporte dos coordenadores/supervisores de enfermagem aos enfermeiros/ equipe de enfermagem	3,23 + 0,57^^*^^	2,75 + 0,62^^*^^	3,18 + 0,57	0,003
Adequação da equipe e de recursos	2,62 + 0,60^^*^^	1,88 + 0,61^^*^^	3,23 + 0,61^^*^^	0,000^^**^^
Relações colegiais entre enfermeiros e médicos	2,90 + 0,58	2,89 + 0,55	2,82 + 0,59	0,787

* ANOVA One-Way com teste de Tukey;

** p ≤ 0,05.

Tal percepção foi reforçada pela narrativa dos participantes na categoria “Um ambiente onde o humano e o técnico se complementam”, principalmente devido à existência de protocolos que asseguram boas práticas e qualificam a assistência, proporcionando avanços no conhecimento e satisfação em trabalhar no hospital.

*Nós temos condições boas de trabalho, nos proporciona que a gente vá atrás de conhecimento, temos colegas qualificados.* (L31-E11)

*Então quando me perguntam se aqui no hospital falta alguma coisa* [para] *a segurança do paciente ou do trabalhador, eu digo que aqui é o mais desenvolvido que eu já trabalhei, porque há preocupação e há um trabalho pra que isso* [segurança do paciente] *esteja presente todos os dias, seja trabalhado todos os dias. Em relação a esse quesito, eu acredito que a nossa instituição tá bem adiantada.* (C36-E14)

Chama atenção na Tabela 1 que a subescala “Adequação da equipe e de recursos” exibiu, na emergência, um escore de 1,88 (dp = 0,61), abaixo do ponto de corte, retratando uma percepção desfavorável do ambiente em relação às unidades de internação clínica-cirúrgica e terapia intensiva (p=0,000), provavelmente embasada pelo número elevado de pacientes atribuídos na escala de trabalho. A preocupação quanto ao dimensionamento de pessoal ficou evidente no discurso dos enfermeiros da emergência na categoria “Qualidade do cuidado e segurança do paciente em um ambiente complexo”, corroborando com o achado numérico. Os enfermeiros manifestaram que o atendimento por demanda espontânea tende a ocasionar lotação excedente e, nesta situação, um aporte inadequado de profissionais expõe os pacientes a riscos e falhas assistenciais.

[...] *porque se você tem o número adequado de paciente, você consegue prestar uma assistência adequada* [...]. (E22-E15)

[...] *a superlotação aqui na emergência é um dos fatores que implica bastante na nossa prática assistencial, nos cuidados com os pacientes, nos riscos* [...] *têm mais riscos de troca de medicação, troca de pacientes, os cuidados que a gente deveria fazer, os mínimos cuidados, às vezes a gente não consegue fazer.* (E9-E16)

Quanto aos atributos de complexidade no ambiente de prática profissional, a aplicação do Questionário de Caracterização da Complexidade evidenciou que os mesmos foram reconhecidos pelos enfermeiros (Tabela 2).

**Tabela 2:** Presença dos atributos de complexidade no ambiente de prática profissional com base no Questionário de Caracterização da Complexidade, Porto Alegre, Rio Grande do Sul, Brasil, 2021

Atributos de complexidade	Unidades de internação clínica-cirúrgica (n=79)	Emergência (n=23)	Terapia intensiva (n=30)	Valor de *p*
Grande número de elementos que interagem dinamicamente	10,99 + 2,07	11,02 + 1,59	12,21 + 1,72	0,011
Grande diversidade de elementos	11,12 + 3,08	11,34 + 2,62	12,28 + 2,42	0,173
Variabilidade inesperada	5,61 + 1,71^^*^^	6,90 + 2,16^^*^^	5,38 + 1,70^^*^^	0,005^^**^^
Resiliência	9,71 + 1,85	9,80 + 1,39	9,90 + 1,60	0,863

* ANOVA One-Way com teste de Tukey;

** p ≤ 0,05.

Nas entrevistas, os exemplos acerca das vivências profissionais na categoria “O olhar dos enfermeiros para a complexidade” ratificaram tal resultado, de modo que o Grande número de elementos que interagem dinamicamente mostrou-se relacionado ao enfermeiro como um elemento central que transita entre várias áreas e equipes, a “Grande diversidade de elementos” à intensa demanda de atividades e a Resiliência atrelada à capacidade adaptativa diante das adversidades, conforme segue:

[...] *o enfermeiro como sendo um elemento central do cuidado, ele tem que interagir com todas as categorias profissionais, gerenciar sua própria equipe, gerenciar o cuidado aos pacientes.* [...] *tem que fazer tudo isso ao mesmo tempo para que o trabalho aconteça de uma forma sincronizada.* (L4-E9)

[...] *é familiar te perguntando coisa, é o paciente querendo saber o resultado de exames,* [...] *se vai embora.* [...] *eu tenho que evoluir. Fora os procedimentos* [...]*. A gente tem uma demanda bem significativa.* (E22-E15)

[...] *a resiliência ela vem na capacidade da gente* [...] *em tu saber se reorganizar e se reestruturar.* (T23-E10)

Quanto à “Variabilidade inesperada”, os escores inferiores a 7 e próximos do extremo “discordo totalmente” na Tabela 2 tendem a expressar que os enfermeiros não reconheceram e/ou reconheceram pouco a presença das características vinculadas a esse atributo. Na comparação entre as áreas (p=0,005), a emergência apresentou uma média maior nesse elemento, o que pode ser justificado pela ausência de controle no setor, visto que as equipes incorporam a variabilidade da demanda como parte do processo. As falas na categoria “O olhar dos enfermeiros para a complexidade”, ao mesmo tempo em que consideraram esse atributo como um traço inerente da rotina assistencial, também trouxeram aspectos relacionados à forma de trabalho nas áreas.

[...] *às vezes as coisas podem estar muito tranquilas e muito estáveis do paciente, da equipe e tudo, daqui a pouco muda tudo* [...]. (T21-E13)

*Variabilidade inesperada, a gente não tem tanto inesperada, em termos de paciente, porque é um ambiente mais controlado* [terapia intensiva]*, é diferente da emergência, tu sabe que tu tem cinco leitos, tem cinco pacientes* [...]. (T17-E5)

Em contrapartida, na associação entre as subescalas da PES-NWI versão brasileira e os atributos de complexidade (Tabela 3), a “Variabilidade inesperada” foi o único atributo que exibiu correlação significativa (p<0,01) moderada e inversa, sugerindo que pode ser reduzida por meio de algumas características presentes no ambiente de prática.

**Tabela 3:** Correlação entre as subescalas da PES-NWI versão brasileira e os atributos do Questionário de Caracterização da Complexidade, Porto Alegre, Rio Grande do Sul, Brasil, 2021

PES-NWI versão brasileira Subescalas	Questionário de Caracterização da Complexidade Atributos			
	Grande número de elementos que interagem dinamicamente	Grande diversidade de elementos	Variabilidade inesperada	Resiliência
Participação dos enfermeiros na discussão dos assuntos hospitalares	0,126	-0,025	-0,376^^*^^	0,100
Fundamentos de enfermagem voltados para a qualidade do cuidado	0,120	-0,031	-0,445^^*^^	0,061
Habilidade, liderança e suporte dos coordenadores/supervisores de enfermagem aos enfermeiros/ equipe de enfermagem	-0,031	-0,188	-0,390^^*^^	-0,001
Adequação da equipe e de recursos	0,039	-0,094	-0,433^^*^^	0,120
Relações colegiais entre enfermeiros e médicos	0,010	-0,110	-0,303^^*^^	0,159

ǂ ANOVA One-Way com teste de Tukey;

* P < 0,01.

Em relação a este achado, na categoria “Um ambiente onde o humano e o técnico se complementam” foi mencionado que um ambiente de prática profissional com elementos favoráveis às atividades do enfermeiro tende a minimizar traços de “Variabilidade inesperada” e contribui para a qualidade do cuidado e segurança do paciente.

[...] *uma equipe que vem trabalhar coesa, que vem trabalhar feliz, que vem trabalhar se sentindo valorizada, é uma equipe que vai dar mais qualidade pro meu paciente, e se, além disso, eu garantir que tenham treinamento adequado, material pra trabalhar, que tenham respeitados os seus direitos, tenham sua folga* [...] *a gente consegue dar qualidade e segurança ao paciente.* (L4-E9)

O mapa conceitual (Figura 1) representa a integração dos resultados quantitativos e qualitativos, tendo como elementos centrais os construtos “ambiente de prática profissional da enfermagem” e “complexidade do SST” - ambos em destaque vermelho, com subescalas (verde) e atributos (amarelo), respectivamente - conectados com as três categorias (azul) e subtemas que emergiram das entrevistas semiestruturadas.

## DISCUSSÃO

O ambiente de prática profissional mostrou-se favorável na percepção dos enfermeiros, visto que as subescalas da PES-NWI versão brasileira exibiram de quatro a cinco domínios acima de 2,5 em todas as áreas. As entrevistas com os participantes reforçaram esse resultado, de modo que o fato de o hospital ser certificado e apresentar protocolos direcionados para práticas assistenciais alicerçadas na qualidade e segurança do paciente tende a influenciar na satisfação laboral dos profissionais e na concepção positiva acerca do próprio trabalho^(20)^. Tal achado, no entanto, difere de estudos recentes em outras realidades brasileiras que utilizaram o mesmo instrumento de mensuração^(21-23)^.

Em contrapartida, a avaliação da subescala “Adequação da equipe e de recursos” da PES-NWI versão brasileira mostrou-se desfavorável na emergência, quando comparada com as unidades de internação clínico-cirúrgica e terapia intensiva, possivelmente em decorrência da proporção de pacientes atribuídos no último plantão versus profissional, que se apresentou díspar. O adequado dimensionamento de pessoal foi mencionado no relato dos participantes dessa área como um aspecto fundamental, com o entendimento de que influencia na qualidade do cuidado e na mitigação de riscos que comprometem a assistência de enfermagem.

O tema da provisão de recursos humanos é uma constante nas pesquisas de enfermagem, principalmente por ser considerado um elemento que interfere diretamente na qualidade do cuidado e na segurança da assistência^(21,24)^, constatação similar ao presente estudo. Assim, a subescala “Adequação da equipe e de recursos” comumente apresenta os piores escores quando se avalia o ambiente de prática^(6,20,25)^, evidência também observada, ainda que somente no setor de emergência. O quantitativo de pacientes acima da capacidade estimada, além de gerar sobrecarga de trabalho, potencializa a ocorrência de incidentes de segurança do paciente, decorrentes da necessidade de adaptações e soluções improvisadas pelo excesso de processos que precisam ser gerenciados pelas equipes^(26)^. A instituição, campo do presente estudo, estimula a notificação dos incidentes e eventos adversos, possibilitando que os erros sejam vistos de maneira sistêmica e como oportunidades de melhoria, desmistificando a cultura punitiva que ainda persiste em muitas instituições. É necessário repensar continuamente esse processo, pois consiste em um recurso que fornece dados relevantes para análises e avaliações, implementação de barreiras, revisões de fluxos assistenciais e gerenciais.

A aplicação do Questionário de Caracterização da Complexidade apontou que os enfermeiros das unidades de internação clínica-cirúrgica, emergência e terapia intensiva reconheceram, no ambiente de prática, a presença das características que compõem os atributos de complexidade “Grande número de elementos que interagem dinamicamente”, “Grande diversidade de elementos” e “Resiliência”, revelando que o trabalho de enfermagem no sistema sociotécnico hospitalar é complexo. Os participantes, nas entrevistas, exemplificaram com situações práticas diárias a presença dos atributos no ambiente, o que fortaleceu tal compreensão. Assim, o grau de dependência dos pacientes, a quantidade de demandas e a necessidade de abarcar múltiplos conhecimentos e habilidades para um trabalho eficiente sustentam a afirmativa de que o setor saúde consiste em um sistema complexo^(2)^. Estudo realizado em unidades de internação clínica-cirúrgica^(27)^ evidenciou que os enfermeiros sentem-se desafiados ao atuarem em cenários com maior pressão, buscando transformar as dificuldades em aprendizado, ainda que estejam empenhados em modificar tal realidade.

Por sua vez, a “Variabilidade inesperada” exibiu escores menores em relação aos demais atributos, sendo associada na narrativa dos enfermeiros às oscilações no estado de saúde dos pacientes, indicando que resquícios de incerteza e imprevisibilidade estão incorporados na rotina de trabalho. Quanto ao escore maior desse item na emergência, é possível que o atendimento por demanda espontânea, ou a denominada porta aberta, tenha influenciado o resultado quantitativo, visto que tal funcionamento inviabiliza a antecipação das equipes no controle dos acontecimentos. Nesse sentido, nas unidades de internação clínica-cirúrgica e terapia intensiva, o ambiente se apresenta mais controlado e, consequentemente, com menos variações.

Ao correlacionar a PES-NWI versão brasileira e o Questionário de Caracterização da Complexidade, os resultados apontaram que menor “Variabilidade inesperada” resulta em um melhor ambiente de prática profissional, ainda que essa seja uma característica inerente ao trabalho de enfermagem. Este achado ficou evidente nas entrevistas, ao passo que, para os enfermeiros, um ambiente com elementos estruturais e relacionais favoráveis ao trabalho contribui para a qualidade do cuidado e segurança do paciente, e estará menos propenso à influência das variações que permeiam o sistema e trazem riscos assistenciais. Isso corrobora estudos recentes que sustentam que um ambiente com características favoráveis consolidadas estará menos propenso à influência de variações^(7,28,29)^.

### Limitações do estudo

Esta pesquisa refere-se a um recorte de tempo específico anterior à pandemia de Coronavírus, realizada em uma única instituição e com amostragem restrita aos profissionais enfermeiros. Além disso, a consistência interna do Questionário de Caracterização da Complexidade mostrou-se baixa, o que pode ser atribuído à menor disseminação e experiência de aplicação do instrumento em comparação à PES-NWI, indicando a necessidade de futuras pesquisas para evidências mais conclusivas sobre sua confiabilidade.

### Contribuições para a área da Enfermagem

Este estudo possibilita a introdução de reflexões acerca da complexidade do trabalho do enfermeiro e dos elementos que propiciam um ambiente de prática favorável à assistência, o que está alinhado com os movimentos sociopolíticos atuais pela valorização profissional da categoria. Ressalta-se ainda que esta pesquisa legitima as recomendações da Organização Mundial da Saúde e de entidades representativas da enfermagem, que estimulam a produção de evidências científicas voltadas para questões relacionadas às condições de trabalho, qualidade do cuidado e segurança do paciente nos cenários de prática.

## CONCLUSÕES

Os resultados do estudo indicaram uma relação entre o ambiente de prática profissional da enfermagem e a complexidade sociotécnica no contexto hospitalar, possibilitando compreender as características das áreas pesquisadas e as implicações para a qualidade do cuidado e segurança dos processos de trabalho. A complexidade mostrou-se incorporada ao ambiente de prática, que, ao apresentar características favoráveis, tende a minimizar a variabilidade inerente à rotina diária e, consequentemente, manter o sistema com maior estabilidade. Destaca-se ainda a importância de produzir pesquisas que permitam identificar pontos favoráveis e/ou fragilidades dos ambientes de prática, visto que os resultados oportunizarão o avanço de estratégias focadas na melhoria da assistência aos pacientes e na satisfação dos profissionais.

## Data Availability

https://doi.org/10.48331/scielodata.LYJ9X6
